# Crystal structures of *trans*-acetyl­dicarbon­yl(η^5^-cyclo­penta­dien­yl)(di­methyl­phenyl­phosphane)molybdenum(II) and *trans*-acetyl­dicarbon­yl(η^5^-cyclo­penta­dien­yl)(ethyl­diphenyl­phosphane)molybdenum(II)

**DOI:** 10.1107/S1600536814020534

**Published:** 2014-09-20

**Authors:** Matthew T. Whited, Gretchen E. Hofmeister, Connor J. Hodges, Laramie T. Jensen, Samuel H. Keyes, Aurapat Ngamnithiporn, Daron E. Janzen

**Affiliations:** a Department of Chemistry, Carleton College, 1 N College St, Northfield, MN 55057, USA; b Department of Chemistry and Biochemistry, St. Catherine University, 2004 Randolph Ave., St. Paul, MN 55105, USA

**Keywords:** crystal structure, phosphine, acet­yl, piano-stool complex, divalent molybdenum

## Abstract

The title compounds, [Mo(C_5_H_5_)(COCH_3_)P(CH_3_)_2_(C_6_H_5_)(CO)_2_], (1), and [Mo(C_5_H_5_)(COCH_3_)P(C_2_H_5_)(C_6_H_5_)_2_)(CO)_2_], (2), have been prepared by phosphine-induced migratory insertion from [Mo(C_5_H_5_)(CO)_3_(CH_3_)]. Both complex mol­ecules exhibit a four-legged piano-stool geometry with *trans*-disposed carbonyl ligands along with Mo—P bond lengths and C—Mo—P angles that reflect the relative steric pressure of the respective phosphine ligand. The structure of compound (1) exhibits a layered arrangement parallel to (100). Within the layers mol­ecules are linked into chains along [001] by non-classical C—H⋯O inter­actions between the acetyl ligand of one mol­ecule and the phenyl and methyl phosphine substituents of another. In the structure of complex (2), a chain motif of centrosymmetrical dimers is found along [010] through C—H⋯O inter­actions.

## Abstract 2

The crystal structures of the title compounds are compared, showing mol­ecular parameters that reflect the relative steric pressure of their respective phosphine ligands. Their supra­molecular properties are distinct but in both cases are organized around short C—H⋯O contacts involving the acetyl ligands.

## Chemical context   

Cyclo­penta­dienylmolybdenum polycarbonyl complexes [Mo(C_5_H_5_)(CO)_*n*_] with ‘piano-stool’ geometries have been studied extensively for their fundamental organometallic reactivity. In particular, alkyl complexes of the form [Mo(C_5_H_5_)(CO)_3_(*R*)] have been studied for their migratory insertion reactivity (Barnett & Treichel, 1967 ▶; Butler *et al.*, 1967 ▶), affording [Mo(C_5_H_5_)(P*R*_3_)(CO)_2_(CO*R*)] acetyl complexes on exposure to phosphine ligands. Although the insertion reaction shows little dependence on the nature of the phosphine, the corresponding deinsertion shows a strong dependence on steric bulk of the phosphine, with bulkier groups giving enhanced deinsertion rates (Barnett, 1969 ▶; Barnett & Pollmann, 1974 ▶).
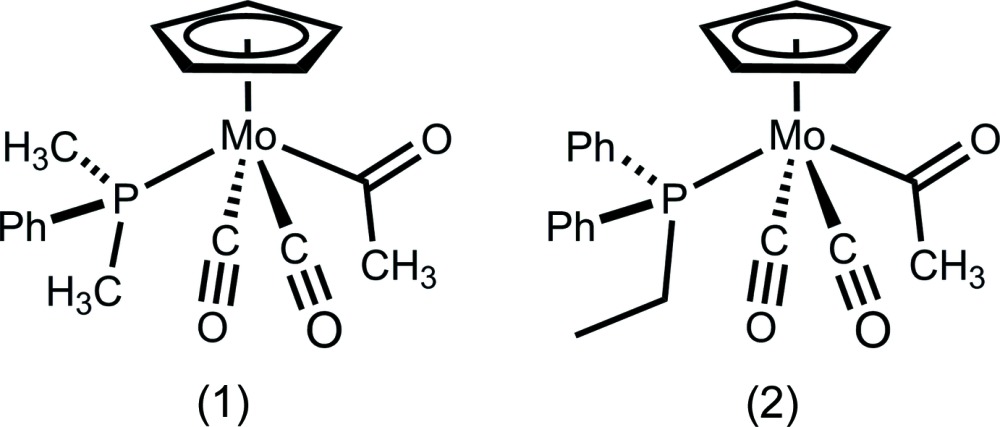


We have developed an inter­est in the solid-state structural properties of a series of piano-stool molybdenum acetyl complexes derived from migratory insertion with various phosphines, with the goal of understanding how modification of the phosphine substituents affects ground-state structure as well as solid-state packing. Recently, we reported an unusual example where orientation of the acetyl group in the solid state can be changed by introduction of furyl substituents on the phosphine ligand (Whited *et al.*, 2013 ▶). In this study, the structures obtained for di­methyl­phenyl­phosphine, [Mo(C_5_H_5_)(P(CH_3_)_2_(C_6_H_5_))(CO)_2_(COCH_3_)] (1), and ethyl­diphenyl­phosphine, [Mo(C_5_H_5_)(P(C_2_H_5_)(C_6_H_5_)_2_))(CO)_2_(COCH_3_)] (2), derivatives are compared.

## Structural commentary   

**Figure 1:**
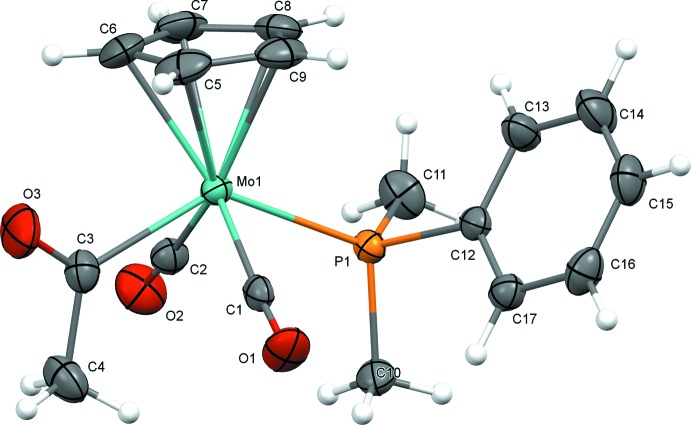
Mol­ecular structure of (1) with displacement ellipsoids drawn at the 50% probability level.

The mol­ecular structures of (1) and (2) are illustrated in Figs. 1 ▶ and 2 ▶. In spite of the somewhat different steric environments provided by the phosphine ligands, the mol­ecular structures are quite similar. Both complexes exhibit a *trans* disposition of carbonyl ligands common for compounds of this class. Complexes (1) and (2) both have structures where the oxygen atom of the acetyl group points toward the cyclo­penta­dienyl (Cp) ring. This orientation is also consistent with the majority of crystal structures of related complexes, with the exception of the recently reported tri(2-fur­yl)phosphine derivative, in which the acetyl group points away from the Cp ring, enabling inter­molecular O⋯H—C inter­actions with the furyl group of a neighboring mol­ecule (Whited *et al.*, 2013 ▶).

Selected geometric parameters for (1) and (2) are presented in Tables 1 ▶ and 2 ▶. The Mo1—P1 bond lengths [2.4535 (9) Å for di­methyl­phenyl­phosphine derivative (1) and 2.4813 (6) Å for ethyl­diphenyl­phosphine derivative (2)] track with the steric bulk of the ligands and are consistent with the previously reported methyl­diphenyl­phosphine complex (Whited *et al.*, 2012 ▶), which exhibits an Mo—P bond length [2.4620 (14) Å] that is inter­mediate between those of (1) and (2). Along with a slightly longer Mo—P distance, the sterically bulkier derivative (2) exhibits a larger C3—Mo1—P1 angle [135.76 (6)°] relative to (1) [131.79 (9)°], again with the methyl­diphenyl­phosphine derivative inter­mediate [132.27 (2)°]. The steric effects of the phosphine ligands observed in the solid state are consistent with findings regarding deca­rbonylation rates for this class of complexes (Barnett & Pollmann, 1974 ▶), where the steric influence of bulkier phosphines enhances the rate of the deca­rbonylation reaction.

## Supra­molecular features   

**Figure 3:**
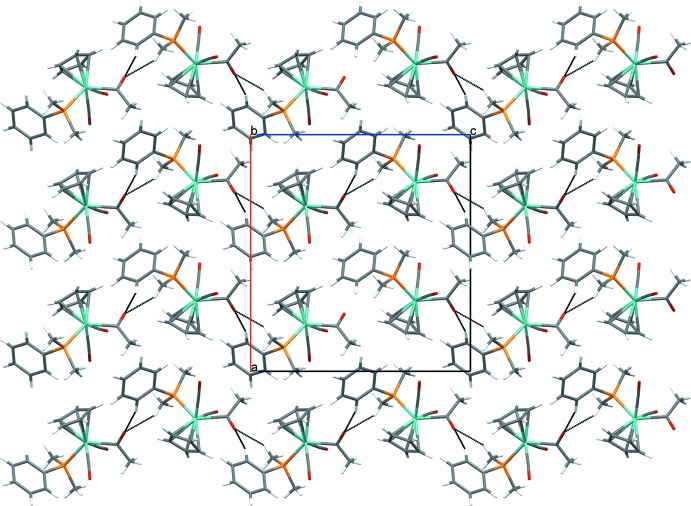
Crystal packing of (1) viewed along [010] showing the layered arrangement parallel to (100). Dashed lines indicate inter­molecular C—H⋯O hydrogen-bonding inter­actions.

The extended structures of (1) and (2) are quite different, but the acetyl oxygen atom (O3) plays an important role in the packing of both structures. For di­methyl­phenyl­phosphine complex (1), there are C—H⋯O hydrogen-bonding inter­actions between O3 of the acetyl carbonyl on one Mo complex and H11*C* from a phosphine methyl substituent (2.45 Å) and H13 from a phenyl group (2.36 Å) on the same phosphine on a neighboring mol­ecule (Table 3 ▶). These short contacts organize the mol­ecules into chains parallel to [001] (Fig. 3 ▶). Additional short contacts (2.40 Å) between O1 of a carbonyl ligand and H15 of a phosphine phenyl substituent within the chains are present. The chains are arranged in layers parallel to (100). In contrast to the closely related methyl­diphenyl­phosphine derivative (Whited *et al.*, 2012 ▶), (1) does not exhibit any π–π inter­actions between the Cp ring and a phosphine phenyl substituent. In contrast, the closest phenyl group is oriented perpendicular to the Cp ring with a distance of 3.00 Å between H17 of the phenyl group and the Cp centroid.

**Figure 4:**
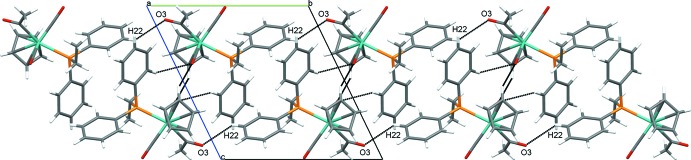
Crystal packing of (2) viewed along [100] showing chains of centrosymmetrical dimers.

**Figure 5:**
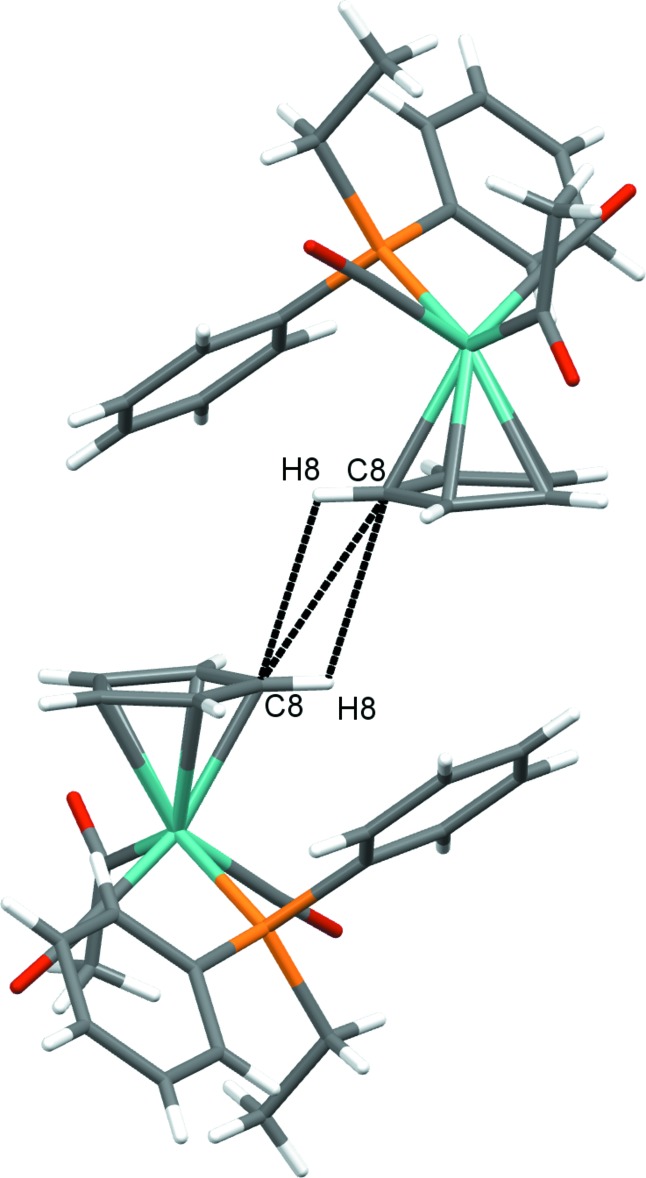
Centrosymmetrical dimers of (2) connected through C8/H8 inter­actions of Cp rings on adjacent mol­ecules.

The supra­molecular organization of ethyl­diphenyl­phosphine derivative (2) is quite different, though it is still partly governed by hydrogen-bonding inter­actions involving O3 of the acetyl group. In this case, short contacts (2.66 Å) between O3 of the acetyl group and H22 of a phosphine phenyl substituent (Table 4 ▶) link the mol­ecules into chains parallel to [010]. An additional set of short contacts between O2 of a carbonyl ligand and H8 from a Cp ring (2.63 Å) and H13 from a phenyl ring (2.71 Å) on an adjacent mol­ecule organize the mol­ecules into centrosymmetrical dimers, joining the unit cells along [010] (Fig. 4 ▶). Finally, another set of centrosymmetrical dimers is formed through short contacts between C8/H8 units on Cp rings of adjacent mol­ecules (Fig. 5 ▶).

## Database survey   

The current version of the Cambridge Structural Database (Version 5.35, updated November 2013; Allen, 2002 ▶) has nine entries corresponding to molybdenum acyl complexes of the general form [Mo(C_5_H_5_)(CO)_2_(P*R*_3_)(CO*R*)], as well as five tungsten complexes with the same ligand types. No chromium complexes with the same ligand set are in the database. The *trans*-dicarbonyl structure, as observed for (1) and (2), is preferred except in cases where the phosphine and acyl ligands are covalently linked, forcing them to be *cis* (Adams *et al.*, 1991 ▶; Mercier *et al.*, 1993 ▶; Yan *et al.*, 2009 ▶). The preference for a *trans* geometry is likely at least partly steric in nature, since the only example with a *cis*-dicarbonyl geometry without linked phosphine and acyl ligands is for a molybdenum formyl with a small tri­methyl­phosphine ligand and a bulky penta­methyl­cyclo­penta­dienyl ligand (Asdar *et al.*, 1989 ▶).

## Synthesis and crystallization   

**CpMo(CO)_3_(CH_3_)**. This compound was prepared by a modification of the method used of Gladysz *et al.* (1979 ▶), as previously reported by Whited & Hofmeister (2014 ▶).

**CpMo(CO)_2_(PMe_2_Ph)(COCH_3_) (1)**. In an inert-atmos­phere glove box, CpMo(CO)_3_(CH_3_) (113 mg, 0.435 mmol) was dissolved in 2 ml aceto­nitrile. In a separate vial, di­methyl­phenyl­phosphine (97.0 mg, 0.702 mmol) was dissolved in 2 ml aceto­nitrile. The vials were combined and the resulting solution was stirred for 1 week. Solvent was removed *in vacuo*, leaving a yellow–orange solid that was triturated with pentane (5 ml) and isolated by filtration to afford the desired product in pure form as a yellow powder (112 mg, 65%). Crystalline material was obtained as yellow–orange prisms by chilling a concentrated diethyl ether solution at 233 K. ^1^H NMR (400 MHz, CDCl_3_): δ 7.67–7.58 (*m*, 2H, Ar–*H*), 7.50–7.41 (*m*, 3H, Ar–*H*), 4.97 (*d*, *J* = 1.1 Hz, 5H, Cp *H*), 2.58 (*s*, 3H, C(O)C*H*_3_), 1.91 (*d*, ^2^ *J*_PH_ = 8.9 Hz, 6H, P(C*H*_3_)_2_). ^13^C{^1^H} NMR (101 MHz, CDCl_3_): δ 267.3 (*d*, ^2^ *J*_PC_ = 13 Hz, –*C*OCH_3_), 237.8 (*d*, ^2^ *J*_PC_ = 24 Hz, –*C*O), 139.2 (*d*, ^1^ *J*_PC_ = 40 Hz, C_ipso_ from *Ph*–P), 130.3 (*d*, ^4^ *J*_PC_ = 2 Hz, C_para_ from *Ph*–P), 129.6 (*d*, ^2^ *J*_PC_ = 10 Hz, C_ortho_ from *Ph*–P), 128.9 (*d*, ^2^ *J*_PC_ = 10 Hz, C_meta_ from *Ph*–P), 96.0 (*s*, Cp ring), 51.7 (*s*, –CO*C*H_3_), 20.0 (*d*, ^1^ *J*_PC_ = 33 Hz, P(*C*H_3_)_2_). ^31^P{^1^H} NMR (162 MHz, CDCl_3_): δ 33.1 (s). IR (CH_2_Cl_2_, NaCl, cm^−1^) ν(CO): 1931, 1846, 1601 (acet­yl).

**CpMo(CO)_2_(PEtPh_2_)(COCH_3_) (2)**. In an inert-atmosphere glove box, CpMo(CO)_3_(CH_3_) (105 mg, 0.404 mmol) was dissolved in 2 ml aceto­nitrile. Ethyl­diphenyl­phosphine (129 mg, 0.602 mmol) was added and the resulting solution was stirred for one week. Solvent was removed *in vacuo*, leaving a yellow solid that was triturated with pentane (5 ml) and isolated by filtration to afford the desired product in pure form as a yellow powder (106 mg, 55%). Crystalline material was obtained as yellow blocks by slow evaporation of diethyl ether from a concentrated solution at ambient temperature. ^1^H NMR (400 MHz, CDCl_3_): δ 7.50–7.42 (*m*, 10H, Ar–*H*), 4.92 (*d*, *J* = 1.2 Hz, 5H, Cp *H*), 2.68 (apparent quint, ^2^ *J*_PH_ = ^3^ *J*_HH_ = 7.8 Hz, 2H, PC*H*_2_CH_3_), 2.63 (*s*, 3H, C(O)C*H*_3_), 1.17 (*dt*, ^3^ *J*_PH_ = 18.0 Hz, ^2^ *J*_PH_ = 7.5 Hz, 3H, PCH_2_C*H*_3_). ^13^C{^1^H} NMR (101 MHz, CDCl_3_): δ 266.4 (*d*, ^2^ *J*_PC_ = 11 Hz, –*C*OCH_3_), 238.5 (*d*, ^2^ *J*_PC_ = 23 Hz, –*C*O), 135.9 (*d*, ^1^ *J*_PC_ = 40 Hz, C_ipso_ from *Ph*–P), 132.1 (*d*, ^2^ *J*_PC_ = 10 Hz, C_ortho_ from *Ph*–P), 130.4 (*d*, ^4^ *J*_PC_ = 2 Hz, C_para_ from *Ph*–P), 128.7 (*d*, ^2^ *J*_PC_ = 9 Hz, C_meta_ from *Ph*–P), 96.5 (*s*, Cp ring), 51.0 (s, –CO*C*H_3_), 26.3 (*d*, ^1^ *J*_PC_ = 32 Hz, P*C*H_2_CH_3_), 9.0 (*d*, ^2^ *J*_PC_ = 2 Hz, PCH_2_ *C*H_3_). ^31^P{^1^H} NMR (162 MHz, CDCl_3_): δ 59.3 (*s*). IR (CH_2_Cl_2_, NaCl, cm^−1^) ν(CO): 1937, 1859, 1610 (acet­yl).

## Refinement   

Crystal data, data collection and structure refinement details are summarized in Table 5 ▶. H-atoms were treated in calculated positions and refined in the riding-model approximation with distances of C—H = 0.95, 1.00 and 0.98 Å for the phenyl, cyclo­penta­dienyl and alkyl groups, respectively, and with *U_iso_*(H) = *k*×*U_eq_*(C), *k* = 1.2 for phenyl and cyclo­penta­dienyl groups and 1.5 for alkyl groups. Methyl group H atoms were allowed to rotate in order to find the best rotameric conformation.

A small number of low-angle reflections [three for (1); six for (2)] were rejected from these high-quality data sets due to the arrangement of the instrument with a conservatively sized beam stop and a fixed-position detector. The large number of reflections in the data sets (and the Fourier-transform relationship of intensities to atoms) ensures that no particular bias was thereby introduced.

## Supplementary Material

Crystal structure: contains datablock(s) 1, 2, general. DOI: 10.1107/S1600536814020534/wm5058sup1.cif

Structure factors: contains datablock(s) 1. DOI: 10.1107/S1600536814020534/wm50581sup2.hkl

Structure factors: contains datablock(s) 2. DOI: 10.1107/S1600536814020534/wm50582sup3.hkl

CCDC references: 1024131, 1024132

Additional supporting information:  crystallographic information; 3D view; checkCIF report

## Figures and Tables

**Figure 2:**
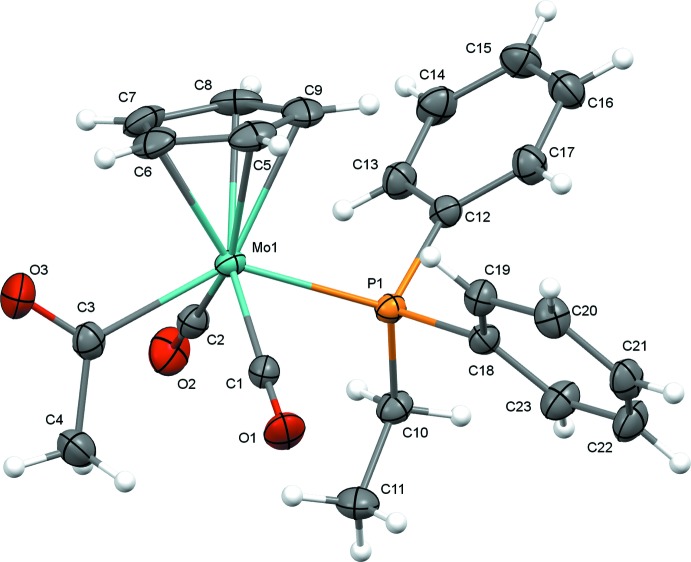
Mol­ecular structure of (2) with displacement ellipsoids drawn at the 50% probability level.

**Table 1:** Selected geometric parameters (Å, °) for (1)

Mo1—P1	2.4535 (9)	Mo1—C2	1.973 (4)
Mo1—C1	1.949 (3)	Mo1—C3	2.251 (4)
			
C1—Mo1—P1	76.95 (9)	C2—Mo1—P1	78.13 (11)
C1—Mo1—C2	106.40 (14)	C2—Mo1—C3	76.05 (15)
C1—Mo1—C3	72.37 (13)	C3—Mo1—P1	131.79 (9)

**Table 2:** Selected geometric parameters (Å, °) for (2)

Mo1—P1	2.4813 (6)	Mo1—C2	1.960 (2)
Mo1—C1	1.979 (2)	Mo1—C3	2.273 (2)
			
C1—Mo1—P1	79.07 (6)	C2—Mo1—C1	106.04 (9)
C1—Mo1—C3	75.46 (8)	C2—Mo1—C3	73.53 (9)
C2—Mo1—P1	79.67 (6)	C3—Mo1—P1	135.76 (6)

**Table 3:** Hydrogen-bond geometry (Å, °) for (1)

*D*—H⋯*A*	*D*—H	H⋯*A*	*D*⋯*A*	*D*—H⋯*A*
C11—H11*C*⋯O3^i^	0.98	2.45	3.344 (5)	152
C13—H13⋯O3^i^	0.95	2.36	3.275 (5)	162

Symmetry code: (i) 
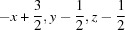
.

**Table 4:** Hydrogen-bond geometry (Å, °) for (2)

*D*—H⋯*A*	*D*—H	H⋯*A*	*D*⋯*A*	*D*—H⋯*A*
C8—H8⋯O2^i^	0.93	2.63	3.414 (3)	142
C13—H13⋯O2^i^	0.93	2.71	3.282 (3)	121
C22—H22⋯O3^ii^	0.93	2.66	3.316 (3)	128

Symmetry codes: (i) 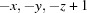; (ii) 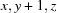.

**Table 5:** Experimental details

	(1)	(2)
Crystal data		
Chemical formula	[Mo(C_5_H_5_)(C_2_H_3_O)(C_8_H_11_P)(CO)_2_]	[Mo(C_5_H_5_)(C_2_H_3_O)(C_14_H_15_P)(CO)_2_]
*M*_r_	398.23	474.32
Crystal system, space group	Orthorhombic, *P* *n* *a*2_1_	Triclinic, *P* 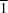
Temperature (K)	173	173
*a*, *b*, *c* (Å)	16.374 (2), 6.8898 (10), 15.208 (2)	8.2451 (8), 11.6132 (11), 12.5265 (12)
α, β, γ (°)	90, 90, 90	63.617 (4), 77.167 (5), 84.671 (6)
*V* (Å^3^)	1715.6 (4)	1047.65 (18)
*Z*	4	2
Radiation type	Mo *K*α	Mo *K*α
μ (mm^−1^)	0.87	0.72
Crystal size (mm)	0.4 × 0.4 × 0.19	0.32 × 0.26 × 0.21

Data collection		
Diffractometer	Rigaku XtaLAB mini	Rigaku XtaLAB mini
Absorption correction	Multi-scan (*REQAB*; Rigaku, 1998 ▶)	Multi-scan (*REQAB*; Rigaku, 1998 ▶)
*T*_min_, *T*_max_	0.707, 0.848	0.712, 0.859
No. of measured, independent and observed [*I* > 2σ(*I*)] reflections	17021, 3923, 3639	11081, 4797, 4365
*R*_int_	0.035	0.029
(sin θ/λ)_max_ (Å^−1^)	0.649	0.649

Refinement		
*R*[*F* ^2^ > 2σ(*F* ^2^)], *wR*(*F* ^2^), *S*	0.021, 0.045, 1.05	0.028, 0.068, 1.09
No. of reflections	3923	4797
No. of parameters	202	255
No. of restraints	1	0
H-atom treatment	H-atom parameters constrained	H-atom parameters constrained
Δρ_max_, Δρ_min_ (e Å^−3^)	0.21, −0.27	0.30, −0.82
Absolute structure	Flack x determined using 1649 quotients [(*I* ^+^)−(*I* ^−^)]/[(*I* ^+^)+(*I* ^−^)] (Parsons *et al.*, 2013 ▶).	–
Absolute structure parameter	0.007 (18)	–

Computer programs: *CrystalClear* (Rigaku, 2011 ▶), *SHELXS* and *SHELXL* (Sheldrick, 2008 ▶), *SIR2008* (Burla *et al.*, 2007 ▶) and *OLEX2* (Dolomanov *et al.*, 2009 ▶).

## supplementary crystallographic information

### Crystal data

**Table:**

[Mo(C_5_H_5_)(C_2_H_3_O)(C_14_H_15_P)(CO)_2_]	*Z* = 2
*M**_r_* = 474.32	*F*(000) = 484
Triclinic, *P*1	*D*_x_ = 1.504 Mg m^−^^3^
*a* = 8.2451 (8) Å	Mo *K*α radiation, λ = 0.71073 Å
*b* = 11.6132 (11) Å	Cell parameters from 10268 reflections
*c* = 12.5265 (12) Å	θ = 3.2–27.6°
α = 63.617 (4)°	µ = 0.72 mm^−^^1^
β = 77.167 (5)°	*T* = 173 K
γ = 84.671 (6)°	Prism, yellow
*V* = 1047.65 (18) Å^3^	0.32 × 0.26 × 0.21 mm

### Data collection

**Table:**

Rigaku XtaLAB mini diffractometer	4365 reflections with *I* > 2σ(*I*)
Detector resolution: 6.849 pixels mm^-1^	*R*_int_ = 0.029
ω scans	θ_max_ = 27.5°, θ_min_ = 3.2°
Absorption correction: multi-scan (*REQAB*; Rigaku, 1998)	*h* = −10→10
*T*_min_ = 0.712, *T*_max_ = 0.859	*k* = −15→15
11081 measured reflections	*l* = −16→16
4797 independent reflections	

### Refinement

**Table:**

Refinement on *F*^2^	Primary atom site location: heavy-atom method
Least-squares matrix: full	Hydrogen site location: inferred from neighbouring sites
*R*[*F*^2^ > 2σ(*F*^2^)] = 0.028	H-atom parameters constrained
*wR*(*F*^2^) = 0.068	*w* = 1/[σ^2^(*F*_o_^2^) + (0.029*P*)^2^ + 0.4738*P*] where *P* = (*F*_o_^2^ + 2*F*_c_^2^)/3
*S* = 1.09	(Δ/σ)_max_ = 0.001
4797 reflections	Δρ_max_ = 0.30 e Å^−^^3^
255 parameters	Δρ_min_ = −0.82 e Å^−^^3^
0 restraints	

### Special details

**Table:**

Geometry. All e.s.d.'s (except the e.s.d. in the dihedral angle between two l.s. planes) are estimated using the full covariance matrix. The cell e.s.d.'s are taken into account individually in the estimation of e.s.d.'s in distances, angles and torsion angles; correlations between e.s.d.'s in cell parameters are only used when they are defined by crystal symmetry. An approximate (isotropic) treatment of cell e.s.d.'s is used for estimating e.s.d.'s involving l.s. planes.

### Fractional atomic coordinates and isotropic or equivalent isotropic displacement parameters (Å^2^)

**Table:**

	*x*	*y*	*z*	*U*_iso_*/*U*_eq_	
Mo1	0.25391 (2)	0.22164 (2)	0.24017 (2)	0.01783 (6)	
P1	0.15696 (6)	0.36677 (5)	0.33857 (5)	0.01874 (11)	
O1	0.2231 (2)	0.48000 (14)	0.01116 (14)	0.0328 (4)	
O2	−0.1013 (2)	0.10837 (16)	0.37595 (17)	0.0379 (4)	
O3	0.2283 (2)	0.07548 (18)	0.09140 (19)	0.0459 (5)	
C1	0.2279 (3)	0.3845 (2)	0.0971 (2)	0.0225 (4)	
C2	0.0285 (3)	0.1541 (2)	0.3233 (2)	0.0248 (4)	
C3	0.1579 (3)	0.1604 (2)	0.1164 (2)	0.0256 (5)	
C4	0.0061 (3)	0.2187 (2)	0.0626 (2)	0.0361 (6)	
H4A	−0.0914	0.1955	0.1265	0.054*	
H4B	−0.0043	0.1869	0.0055	0.054*	
H4C	0.0177	0.3106	0.0217	0.054*	
C5	0.5510 (3)	0.2184 (2)	0.2032 (2)	0.0313 (5)	
H5	0.6164	0.2873	0.1426	0.038*	
C6	0.4935 (3)	0.1161 (2)	0.1898 (2)	0.0319 (5)	
H6	0.5130	0.1063	0.1183	0.038*	
C7	0.4005 (3)	0.0304 (2)	0.3044 (2)	0.0331 (5)	
H7	0.3493	−0.0456	0.3213	0.040*	
C8	0.4000 (3)	0.0813 (2)	0.3878 (2)	0.0321 (5)	
H8	0.3485	0.0450	0.4696	0.039*	
C9	0.4922 (3)	0.1978 (2)	0.3243 (2)	0.0309 (5)	
H9	0.5107	0.2517	0.3575	0.037*	
C10	−0.0688 (3)	0.3899 (2)	0.3745 (2)	0.0247 (4)	
H10A	−0.1210	0.3075	0.4308	0.030*	
H10B	−0.0899	0.4456	0.4151	0.030*	
C11	−0.1500 (3)	0.4481 (2)	0.2632 (2)	0.0347 (5)	
H11A	−0.1027	0.5316	0.2084	0.052*	
H11B	−0.2675	0.4560	0.2887	0.052*	
H11C	−0.1307	0.3934	0.2226	0.052*	
C12	0.2069 (3)	0.30616 (19)	0.49000 (19)	0.0219 (4)	
C13	0.1367 (3)	0.1892 (2)	0.5807 (2)	0.0273 (5)	
H13	0.0644	0.1458	0.5636	0.033*	
C14	0.1734 (3)	0.1371 (2)	0.6958 (2)	0.0317 (5)	
H14	0.1254	0.0593	0.7554	0.038*	
C15	0.2814 (3)	0.2006 (2)	0.7222 (2)	0.0338 (5)	
H15	0.3055	0.1660	0.7996	0.041*	
C16	0.3534 (3)	0.3154 (2)	0.6333 (2)	0.0347 (5)	
H16	0.4268	0.3577	0.6508	0.042*	
C17	0.3166 (3)	0.3686 (2)	0.5174 (2)	0.0284 (5)	
H17	0.3656	0.4461	0.4580	0.034*	
C18	0.2429 (3)	0.53126 (19)	0.26053 (19)	0.0216 (4)	
C19	0.4009 (3)	0.5528 (2)	0.18706 (19)	0.0245 (4)	
H19	0.4597	0.4851	0.1765	0.029*	
C20	0.4719 (3)	0.6756 (2)	0.1291 (2)	0.0272 (5)	
H20	0.5786	0.6889	0.0813	0.033*	
C21	0.3841 (3)	0.7775 (2)	0.1424 (2)	0.0294 (5)	
H21	0.4309	0.8595	0.1028	0.035*	
C22	0.2268 (3)	0.7569 (2)	0.2148 (2)	0.0334 (5)	
H22	0.1674	0.8253	0.2235	0.040*	
C23	0.1566 (3)	0.6340 (2)	0.2749 (2)	0.0306 (5)	
H23	0.0514	0.6205	0.3248	0.037*	

### Atomic displacement parameters (Å^2^)

**Table:**

	*U*^11^	*U*^22^	*U*^33^	*U*^12^	*U*^13^	*U*^23^
Mo1	0.01765 (10)	0.01435 (9)	0.01900 (10)	0.00154 (6)	−0.00451 (7)	−0.00496 (7)
P1	0.0185 (2)	0.0163 (2)	0.0196 (3)	0.00010 (19)	−0.0039 (2)	−0.0062 (2)
O1	0.0459 (10)	0.0201 (8)	0.0262 (8)	0.0016 (7)	−0.0129 (8)	−0.0023 (7)
O2	0.0284 (9)	0.0325 (9)	0.0484 (11)	−0.0103 (7)	0.0027 (8)	−0.0168 (8)
O3	0.0520 (12)	0.0396 (10)	0.0655 (13)	0.0108 (9)	−0.0235 (10)	−0.0368 (10)
C1	0.0230 (10)	0.0208 (10)	0.0270 (11)	0.0015 (8)	−0.0070 (9)	−0.0126 (9)
C2	0.0264 (11)	0.0180 (10)	0.0297 (11)	0.0006 (8)	−0.0084 (9)	−0.0089 (9)
C3	0.0294 (11)	0.0214 (11)	0.0252 (11)	−0.0043 (9)	−0.0052 (9)	−0.0087 (9)
C4	0.0410 (14)	0.0355 (13)	0.0378 (14)	0.0009 (11)	−0.0194 (12)	−0.0161 (11)
C5	0.0166 (10)	0.0304 (12)	0.0373 (13)	0.0045 (9)	−0.0036 (9)	−0.0077 (10)
C6	0.0258 (12)	0.0348 (13)	0.0348 (13)	0.0148 (10)	−0.0068 (10)	−0.0173 (11)
C7	0.0329 (13)	0.0183 (11)	0.0421 (14)	0.0091 (9)	−0.0128 (11)	−0.0074 (10)
C8	0.0244 (11)	0.0354 (13)	0.0247 (11)	0.0100 (10)	−0.0098 (9)	−0.0022 (10)
C9	0.0190 (11)	0.0379 (13)	0.0396 (13)	0.0083 (9)	−0.0118 (10)	−0.0191 (11)
C10	0.0203 (10)	0.0230 (11)	0.0284 (11)	0.0009 (8)	−0.0025 (9)	−0.0105 (9)
C11	0.0265 (12)	0.0369 (13)	0.0442 (15)	0.0082 (10)	−0.0157 (11)	−0.0184 (12)
C12	0.0228 (10)	0.0208 (10)	0.0213 (10)	0.0024 (8)	−0.0051 (8)	−0.0086 (8)
C13	0.0321 (12)	0.0214 (11)	0.0272 (11)	−0.0026 (9)	−0.0069 (10)	−0.0086 (9)
C14	0.0400 (13)	0.0220 (11)	0.0260 (12)	0.0008 (10)	−0.0070 (10)	−0.0041 (9)
C15	0.0439 (14)	0.0332 (13)	0.0254 (12)	0.0074 (11)	−0.0163 (11)	−0.0110 (10)
C16	0.0403 (14)	0.0349 (13)	0.0348 (13)	−0.0014 (11)	−0.0171 (11)	−0.0157 (11)
C17	0.0320 (12)	0.0259 (11)	0.0267 (11)	−0.0045 (9)	−0.0063 (10)	−0.0100 (9)
C18	0.0246 (10)	0.0175 (10)	0.0215 (10)	−0.0004 (8)	−0.0073 (8)	−0.0062 (8)
C19	0.0267 (11)	0.0219 (11)	0.0255 (11)	−0.0002 (8)	−0.0046 (9)	−0.0111 (9)
C20	0.0281 (11)	0.0283 (12)	0.0234 (11)	−0.0072 (9)	−0.0003 (9)	−0.0105 (9)
C21	0.0398 (13)	0.0193 (11)	0.0269 (12)	−0.0065 (9)	−0.0080 (10)	−0.0063 (9)
C22	0.0392 (14)	0.0199 (11)	0.0418 (14)	0.0019 (10)	−0.0078 (11)	−0.0146 (10)
C23	0.0297 (12)	0.0240 (11)	0.0347 (13)	0.0004 (9)	−0.0020 (10)	−0.0118 (10)

### Geometric parameters (Å, º)

**Table:**

Mo1—P1	2.4813 (6)	C10—H10A	0.9700
Mo1—C1	1.979 (2)	C10—H10B	0.9700
Mo1—C2	1.960 (2)	C10—C11	1.530 (3)
Mo1—C3	2.273 (2)	C11—H11A	0.9600
Mo1—C5	2.390 (2)	C11—H11B	0.9600
Mo1—C6	2.342 (2)	C11—H11C	0.9600
Mo1—C7	2.321 (2)	C12—C13	1.398 (3)
Mo1—C8	2.340 (2)	C12—C17	1.393 (3)
Mo1—C9	2.368 (2)	C13—H13	0.9300
P1—C10	1.839 (2)	C13—C14	1.386 (3)
P1—C12	1.836 (2)	C14—H14	0.9300
P1—C18	1.841 (2)	C14—C15	1.385 (3)
O1—C1	1.158 (3)	C15—H15	0.9300
O2—C2	1.158 (3)	C15—C16	1.380 (3)
O3—C3	1.223 (3)	C16—H16	0.9300
C3—C4	1.514 (3)	C16—C17	1.395 (3)
C4—H4A	0.9600	C17—H17	0.9300
C4—H4B	0.9600	C18—C19	1.389 (3)
C4—H4C	0.9600	C18—C23	1.392 (3)
C5—H5	0.9300	C19—H19	0.9300
C5—C6	1.408 (3)	C19—C20	1.396 (3)
C5—C9	1.403 (3)	C20—H20	0.9300
C6—H6	0.9300	C20—C21	1.384 (3)
C6—C7	1.422 (3)	C21—H21	0.9300
C7—H7	0.9300	C21—C22	1.379 (3)
C7—C8	1.410 (4)	C22—H22	0.9300
C8—H8	0.9300	C22—C23	1.395 (3)
C8—C9	1.417 (3)	C23—H23	0.9300
C9—H9	0.9300		
			
C1—Mo1—P1	79.07 (6)	C6—C7—Mo1	73.06 (12)
C1—Mo1—C3	75.46 (8)	C6—C7—H7	126.2
C1—Mo1—C5	97.88 (8)	C8—C7—Mo1	73.12 (13)
C1—Mo1—C6	109.55 (9)	C8—C7—C6	107.7 (2)
C1—Mo1—C7	144.10 (9)	C8—C7—H7	126.2
C1—Mo1—C8	153.01 (9)	Mo1—C8—H8	120.3
C1—Mo1—C9	118.12 (9)	C7—C8—Mo1	71.68 (12)
C2—Mo1—P1	79.67 (6)	C7—C8—H8	126.2
C2—Mo1—C1	106.04 (9)	C7—C8—C9	107.6 (2)
C2—Mo1—C3	73.53 (9)	C9—C8—Mo1	73.60 (12)
C2—Mo1—C5	155.94 (9)	C9—C8—H8	126.2
C2—Mo1—C6	129.67 (9)	Mo1—C9—H9	121.0
C2—Mo1—C7	99.26 (9)	C5—C9—Mo1	73.70 (13)
C2—Mo1—C8	99.09 (9)	C5—C9—C8	108.7 (2)
C2—Mo1—C9	128.81 (9)	C5—C9—H9	125.7
C3—Mo1—P1	135.76 (6)	C8—C9—Mo1	71.38 (12)
C3—Mo1—C5	110.95 (8)	C8—C9—H9	125.7
C3—Mo1—C6	82.22 (8)	P1—C10—H10A	108.7
C3—Mo1—C7	88.08 (8)	P1—C10—H10B	108.7
C3—Mo1—C8	122.06 (9)	H10A—C10—H10B	107.6
C3—Mo1—C9	139.73 (8)	C11—C10—P1	114.02 (16)
C5—Mo1—P1	107.89 (6)	C11—C10—H10A	108.7
C6—Mo1—P1	140.94 (6)	C11—C10—H10B	108.7
C6—Mo1—C5	34.59 (8)	C10—C11—H11A	109.5
C6—Mo1—C9	57.61 (8)	C10—C11—H11B	109.5
C7—Mo1—P1	131.14 (7)	C10—C11—H11C	109.5
C7—Mo1—C5	58.24 (8)	H11A—C11—H11B	109.5
C7—Mo1—C6	35.51 (8)	H11A—C11—H11C	109.5
C7—Mo1—C8	35.20 (9)	H11B—C11—H11C	109.5
C7—Mo1—C9	58.20 (9)	C13—C12—P1	118.36 (16)
C8—Mo1—P1	96.21 (7)	C17—C12—P1	123.11 (16)
C8—Mo1—C5	57.94 (8)	C17—C12—C13	118.5 (2)
C8—Mo1—C6	58.45 (9)	C12—C13—H13	119.5
C8—Mo1—C9	35.03 (8)	C14—C13—C12	120.9 (2)
C9—Mo1—P1	84.34 (6)	C14—C13—H13	119.5
C9—Mo1—C5	34.29 (8)	C13—C14—H14	120.0
C10—P1—Mo1	117.10 (7)	C15—C14—C13	120.0 (2)
C10—P1—C18	103.97 (10)	C15—C14—H14	120.0
C12—P1—Mo1	112.60 (7)	C14—C15—H15	120.1
C12—P1—C10	100.72 (10)	C16—C15—C14	119.8 (2)
C12—P1—C18	102.90 (9)	C16—C15—H15	120.1
C18—P1—Mo1	117.35 (7)	C15—C16—H16	119.8
O1—C1—Mo1	175.79 (19)	C15—C16—C17	120.4 (2)
O2—C2—Mo1	176.56 (19)	C17—C16—H16	119.8
O3—C3—Mo1	120.13 (17)	C12—C17—C16	120.3 (2)
O3—C3—C4	116.7 (2)	C12—C17—H17	119.8
C4—C3—Mo1	123.19 (16)	C16—C17—H17	119.8
C3—C4—H4A	109.5	C19—C18—P1	118.76 (16)
C3—C4—H4B	109.5	C19—C18—C23	118.97 (19)
C3—C4—H4C	109.5	C23—C18—P1	122.26 (17)
H4A—C4—H4B	109.5	C18—C19—H19	119.8
H4A—C4—H4C	109.5	C18—C19—C20	120.4 (2)
H4B—C4—H4C	109.5	C20—C19—H19	119.8
Mo1—C5—H5	122.7	C19—C20—H20	119.9
C6—C5—Mo1	70.86 (12)	C21—C20—C19	120.2 (2)
C6—C5—H5	126.1	C21—C20—H20	119.9
C9—C5—Mo1	72.01 (13)	C20—C21—H21	120.2
C9—C5—H5	126.1	C22—C21—C20	119.7 (2)
C9—C5—C6	107.7 (2)	C22—C21—H21	120.2
Mo1—C6—H6	119.9	C21—C22—H22	119.8
C5—C6—Mo1	74.56 (13)	C21—C22—C23	120.3 (2)
C5—C6—H6	125.9	C23—C22—H22	119.8
C5—C6—C7	108.3 (2)	C18—C23—C22	120.4 (2)
C7—C6—Mo1	71.43 (12)	C18—C23—H23	119.8
C7—C6—H6	125.9	C22—C23—H23	119.8
Mo1—C7—H7	119.5		
			
Mo1—P1—C10—C11	−60.67 (18)	C9—C5—C6—C7	−1.0 (2)
Mo1—P1—C12—C13	−62.49 (18)	C10—P1—C12—C13	63.03 (19)
Mo1—P1—C12—C17	114.86 (18)	C10—P1—C12—C17	−119.61 (19)
Mo1—P1—C18—C19	−28.34 (19)	C10—P1—C18—C19	−159.42 (17)
Mo1—P1—C18—C23	153.15 (17)	C10—P1—C18—C23	22.1 (2)
Mo1—C5—C6—C7	−63.95 (15)	C12—P1—C10—C11	176.90 (17)
Mo1—C5—C9—C8	63.24 (15)	C12—P1—C18—C19	95.88 (18)
Mo1—C6—C7—C8	−65.44 (15)	C12—P1—C18—C23	−82.6 (2)
Mo1—C7—C8—C9	−65.34 (15)	C12—C13—C14—C15	−0.3 (4)
Mo1—C8—C9—C5	−64.74 (16)	C13—C12—C17—C16	−0.6 (3)
P1—C12—C13—C14	178.29 (18)	C13—C14—C15—C16	−0.5 (4)
P1—C12—C17—C16	−177.99 (18)	C14—C15—C16—C17	0.6 (4)
P1—C18—C19—C20	−178.29 (17)	C15—C16—C17—C12	−0.1 (4)
P1—C18—C23—C22	179.50 (18)	C17—C12—C13—C14	0.8 (3)
C5—C6—C7—Mo1	66.00 (15)	C18—P1—C10—C11	70.56 (18)
C5—C6—C7—C8	0.6 (2)	C18—P1—C12—C13	170.22 (17)
C6—C5—C9—Mo1	−62.23 (15)	C18—P1—C12—C17	−12.4 (2)
C6—C5—C9—C8	1.0 (2)	C18—C19—C20—C21	−1.2 (3)
C6—C7—C8—Mo1	65.40 (15)	C19—C18—C23—C22	1.0 (3)
C6—C7—C8—C9	0.1 (2)	C19—C20—C21—C22	0.9 (3)
C7—C8—C9—Mo1	64.07 (15)	C20—C21—C22—C23	0.3 (4)
C7—C8—C9—C5	−0.7 (2)	C21—C22—C23—C18	−1.3 (4)
C9—C5—C6—Mo1	62.98 (15)	C23—C18—C19—C20	0.3 (3)

### Hydrogen-bond geometry (Å, º)

**Table:**

*D*—H···*A*	*D*—H	H···*A*	*D*···*A*	*D*—H···*A*
C8—H8···O2^i^	0.93	2.63	3.414 (3)	142
C13—H13···O2^i^	0.93	2.71	3.282 (3)	121
C22—H22···O3^ii^	0.93	2.66	3.316 (3)	128

Symmetry codes: (i) −*x*, −*y*, −*z*+1; (ii) *x*, *y*+1, *z*.
